# Editorial: Plant secondary metabolic regulation and engineering

**DOI:** 10.3389/fpls.2023.1174717

**Published:** 2023-04-20

**Authors:** Fangyuan Zhang, Xueqing Fu, Yongliang Liu

**Affiliations:** ^1^ Integrative Science Center of Germplasm Creation in Western China (Chongqing) Science City & Southwest University, School of Life Sciences, Southwest University, Chongqing, China; ^2^ School of Design, Shanghai Jiao Tong University, Shanghai, China; ^3^ Department of Plant and Soil Sciences and Kentucky Tobacco Research and Development Center, University of Kentucky, Lexington, KY, United States

**Keywords:** plant secondary metabolites, metabolic biosynthesis, metabolic regulation, metabolic engineering, transcription factor

## Introduction

Plant secondary metabolites (PSMs), or plant specialized metabolites attract great interest they are unique sources of drugs, nutrients, agrochemicals, and chemical additives (Kabera et al., 2014; Tiwari and Rana, 2015). PSMs include several major groups such as phenolics, terpenes, and nitrogen-containing compounds, and they play vital roles in coping with abiotic and biotic stresses (Kaushik et al., 2021; Elshafie et al., 2023). Due to the diversity of PSMs in different plant species, biosynthesis and regulation of PSMs are not fully elucidated. Moreover, most of the PSMs are low in content and metabolic engineering has been used to boost the production of valuable PSMs (Verpoorte and Memelink, 2002; Zheng et al., 2023). This Research Topic presents the most recent advances in 26 publications, including 4 reviews and 22 research articles, contributed by 197 authors. The aim of this topic is to strengthen our understanding of the biosynthesis and regulation of PSMs in non-model plants, especially medicinal plants, and provide efficient routes to elevate the production of valuable PSMs. Here we summarize these papers according to the classification of contributions, mainly including the identification of key pathway genes of PSMs, the elucidation of regulatory mechanisms, and the successful metabolic engineering of several valuable PSMs.

## Identification of key pathway genes of PSMs

Although the value of specific PSMs, their biosynthetic and metabolic pathways are not fully illustrated. In this topic, several candidate genes for the biosynthesis of diterpenoids (ATDs) and phenolic acids (PAs) were identified in the medicinal plant *Salvia apiana* and its close species *S. miltiorrhiza* (Hu et al.). In this study, comparative metabolome and transcriptome analyses between *S. apiana* roots and leaves, and between two species revealed that four cytochromes (CYPs) and clade VII laccases might contribute to the biosynthesis of specific ATDs and PAs, respectively. In another study, two borneol acetyltransferases (BAT), WvBAT3 and WvBAT4, catalyzing the last step of bornyl acetate (BA) biosynthesis were characterized in *Wurfbainia villosa* (Liang et al.). BA is an aromatic monoterpene ester mostly accumulated in the seeds of *W. villosa*. These two BATs presented *in vitro* catalytic efficiency on the substrates of BA, and their gene expression patterns well correlate with the distribution of BA. In *Trigonella foenum-graecum*, Tf3SGT2 was identified as a steroid-specific UDP-glucose 3-O-glucosyltransferase that involves in steroidal saponin biosynthesis (Gao et al.). *In vitro* enzyme assay verified the catalytic activity of Tf3SGT2. Furthermore, RNA interference (RNAi) of *Tf3SGT2* in the hairy roots of *Trigonella foenum-graecum* confirmed the involvement of Tf3SGT2 in steroidal saponin biosynthesis. In another study, DoCCD1 was characterized involving in the biosynthesis of β-ionone in *Dendrobium officinale* (Wang et al.). In both *Escerichia coli* cells and *Nicotiana benthamiana* leaves which contain carotenoid precursors, expression of DoCCD1 resulted in the production of β-ionone. These findings contribute to our understanding of the biosynthetic and metabolic pathways of PSMs and provide a basis for the elucidation of regulatory mechanisms and metabolic engineering of PSMs.

## Elucidation of regulatory mechanisms of PSMs

The Biosynthesis of PSMs is commonly regulated at the transcriptional level by transcription factors (TFs). The gene expression, protein abundance, localization, or trans-activities of the TFs could be modulated by various stimuli and signaling (Vom Endt et al., 2002). In this topic, an R2R3-MYB TF TcMYB29a was identified to regulate taxol biosynthesis in *Taxus chinensis* (Cao et al.). Overexpression of *TcMYB29a* in *T. chinensis* cell suspension cultures led to an increased accumulation of taxol, and upregulated expression of several taxol biosynthetic genes. Moreover, the expression of *TcMYB29a* was strongly enhanced by the treatment of Abscisic acid (ABA), which also induced the production of taxol. In another study, a bHLH TF AabHLH112 was characterized to positively regulate the biosynthesis of three kinds of sesquiterpenes, β-caryophyllene, epi-cedrol, and β -farnesene in *Artemisia annua* (Xiang et al.). AabHLH112 directly binds to the E-box (CANNTG) motifs in the promoters of the biosynthetic genes of these three sesquiterpenes. Exogenous methyl jasmonate (MeJA) enhanced the expression of *AabHLH112*, the biosynthetic genes as well as the contents of sesquiterpenes. In apples, a B-box protein, MdBBX21 was identified to positively involve in the light-induced biosynthesis of anthocyanins in the fruit peel of red apples (Zhang et al.). Overexpression of *MdBBX21* in *Arabidopsis* and apple calli under light increased anthocyanin accumulation. Moreover, the interaction of MdBBX21 and another TF MdHY5 significantly increased their trans-activation on the promoter of a target gene. In another study, a R2R3-MYB TF, NtMIXTA1, was characterized to involve in glandular trichomes (GTs) development in the medicinal plant *Nepeta tenuifolia* (Zhou et al.). GTs are the primary storage organ for monoterpenes in *N. tenuifolia.* Knock-down of *NtMIXTA1* resulted in lower GT density, a significant reduction in monoterpene concentration, and the decreased expression of genes related to monoterpene biosynthesis. The findings enrich the the transcriptional regulatory network of PSMs.

## Metabolic engineering of valuable PSMs

Due to the low concentration of valuable PSMs in plants, metabolic engineering has long been one of the most efficient approaches utilized to boost the accumulation of specific PSMs (Courdavault et al., 2021). In this topic, an *Arabidopsis* MYB-type TF AtMYB12 was overexpressed in licorice (*Glycyrrhiza inflata*) hairy roots and induced the accumulation of total flavonoids as well as the specific licochalcones, licochalcone a (LCA) and echinatin (Wu et al.). Transcriptome analyses of the *AtMYB12*-overexpressing hairy roots implied that the carbohydrate metabolism was likely reprogrammed to increase carbon flux into flavonoid biosynthesis. In another study, a *Salvia miltiorrhiza* GRAS TF *SmSCR1* was overexpressed in *S*. *miltiorrhiza* hairy roots and significantly induced the accumulation of tanshinone (Zhou et al.). In Tartary Buckwheat (*Fagopyrum tataricum*), FtMYB45 is an R2R3-type MYB TF that negatively regulates flavonoid biosynthesis. Knock-out of *FtMYB45* resulted in an increased accumulation of rutin, catechin, and other flavonoids (Wen et al.). Wild tomato species *Solanum habrochaites* produce various sesquiterpenes in the GTs for herbivore defense. Overexpression of a *prenyl transferase gene* and a *terpene synthase gene* in tomato leaves led to an increased accumulation of sesquiterpenes and also enhanced resistance to pests (Wang et al.). Deguchi et al. developed the vacuum agroinfiltration method to increase total CBD content and reduce the total THC content through transiently expressing *CBDAS* gene and silencing *THCAS* gene, respectively. The study suggests that metabolic engineering is an effective strategy to increase the accumulation of valuable PSMs.

PSMs have attracted the attention because of their great economic value, healthcare value and medical value. To improve the production of PSMs, the researchers focus on identification of key pathway genes and understanding regulatory mechanisms of PSMs. This Research Topic is a timely collection of advanced studies on plant secondary metabolic regulation and engineering. Despite further studies are needed, we hope that this Research Topic offers some important insight into this research area.

## Author contributions

FZ, XF, and YL have made a substantial, direct, and intellectual contribution to the work, and approved it for publication in Frontiers in Plant Science.
